# Supplementary material for: Seminoma and Embryonal Carcinoma Footprints Identified by Analysis of Integrated Genome-Wide Epigenetic and Expression Profiles of Germ Cell Cancer Cell Lines
Source: PLoS One. 2014 Jun 2;9(6):e98330. doi: 10.1371/journal.pone.0098330 (PMC4041891; doi:10.1371/journal.pone.0098330)
Supplement: File S1 — ZIP file containing DMRforPairs output for significant regions. Please start from the html files. (ZIP) [file pone.0098330.s008.zip › figures/24221.pdf]

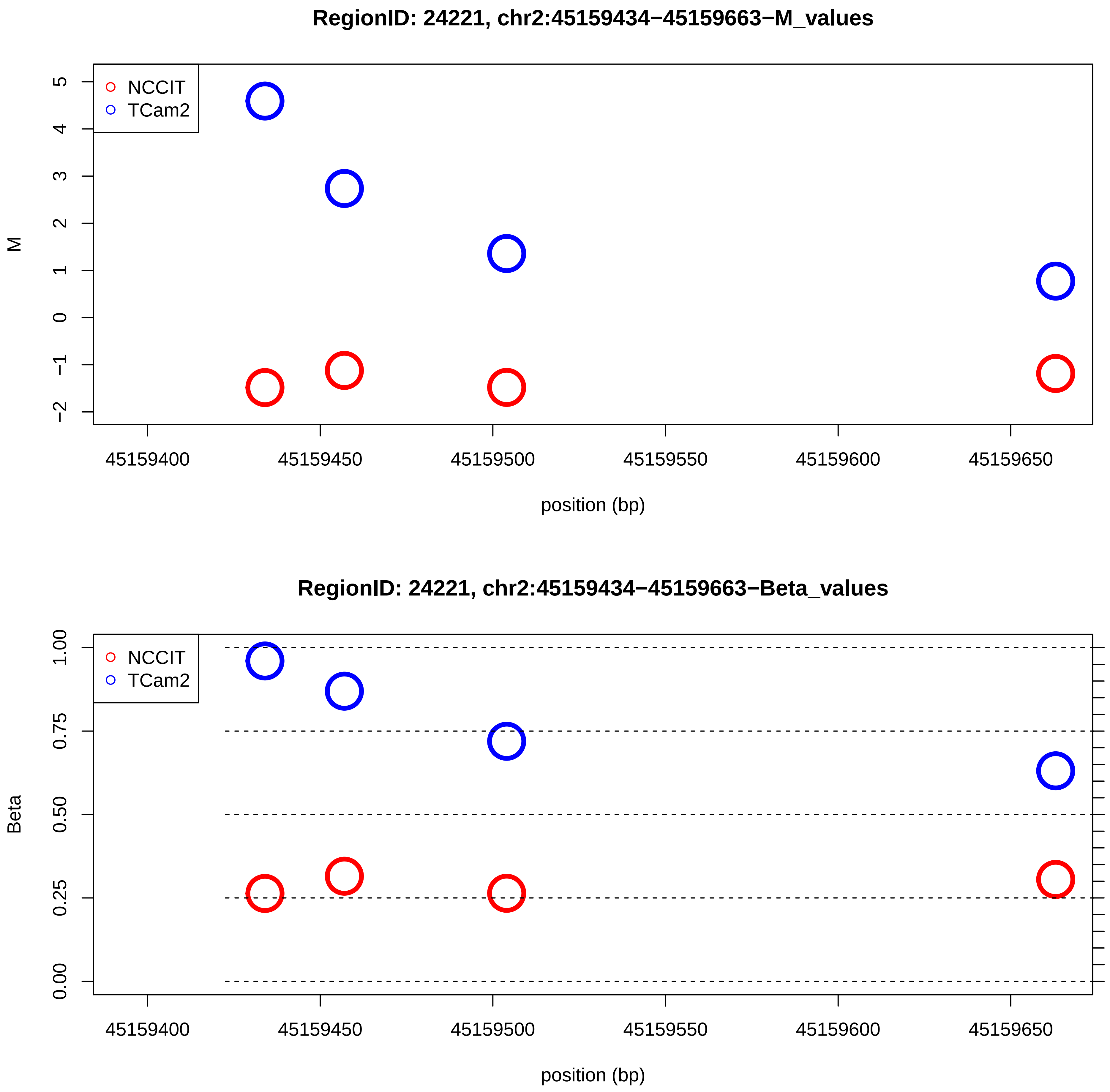
RegionID: 24221, chr2:45159434−45159663−M_values
NCCIT
TCam2
M
position (bp)
45159400
45159450
45159500
45159550
45159600
45159650
RegionID: 24221, chr2:45159434−45159663−Beta_values
NCCIT
TCam2
Beta
position (bp)
